# Enhanced Near-Infrared-Excitable Organic Afterglow Nanoparticles for Deep-Tissue Multimodal Imaging via Singlet Oxygen-Mediated Energy Transfer

**DOI:** 10.34133/research.0834

**Published:** 2025-08-14

**Authors:** Yuzhen Yu, Zhe Li, Shiyi Liao, Baoli Yin, Qingpeng Zhang, Jiaqi Fu, Cheng Zhang, Ying Zhou, Guosheng Song

**Affiliations:** State Key Laboratory of Chemo and Biosensing, College of Chemistry and Chemical Engineering, Hunan University, Changsha 410082, China.

## Abstract

Afterglow imaging offers exceptional signal-to-background ratios (SBRs) by circumventing real-time excitation and autofluorescence, yet conventional systems rely on visible-light excitation, limiting tissue penetration and signal intensity. Here, we report near-infrared-excitable organic afterglow nanoparticles (NOANPs) that leverage singlet oxygen (^1^O_2_)-mediated energy transfer to achieve prolonged, high-intensity emission with minimal photobleaching. The nanoparticles integrate a near-infrared-photoactive sensitizer (NAM-0), which generates abundant ^1^O_2_ under 808-nm laser excitation, and a triplenet-anthracene derivative (TD) as the afterglow substrate, which converts ^1^O_2_ into sustained luminescence. Co-encapsulation via one-step nanocoprecipitation ensures proximity between NAM-0 and TD, enabling efficient energy transfer and yielding exceptional afterglow brightness (>10^9^ photons/s) at ultralow concentrations (10 μg/ml). NOANPs enable deep-tissue imaging (up to 3.0 cm ex vivo) by synergizing the superior penetration of near-infrared light with organic afterglow chemistry. The nanoparticles uniquely support three imaging modes: fluorescence, white light-activated afterglow, and near-infrared-triggered afterglow, which were validated in orthotopic murine models of pancreatic cancer and glioma. By synergizing near-infrared excitation with organic afterglow chemistry, this work overcomes longstanding limitations in penetration depth of excitation light, offering a versatile tool for precision imaging.

## Introduction

Biomedical imaging technologies, despite their transformative role in diagnostics and research, are constrained by inherent trade-offs [1–12]. Modalities like magnetic resonance imaging (MRI) suffer from limited sensitivity, computed tomography (CT) relies on ionizing radiation, ultrasound is susceptible to gas interference, and fluorescence imaging grapples with tissue autofluorescence [13]. Afterglow imaging, which eliminates the need for real-time excitation to reduce background noise and improve the signal-to-background ratio (SBR), has emerged as a promising imaging modality [14–16]. This technique leverages materials that store excitation energy and emit light persistently post-stimulation, enabling high-contrast visualization of biological targets [17–26]. However, the clinical translation of afterglow imaging remains hindered by critical limitations in existing materials and excitation strategies [27].

Traditional afterglow systems rely on inorganic materials [e.g., ZnGa_2(1–*x*)_ Cr_2*x*_O_4_, and NaYbF_4_] [24,25,28–34], in which trapped electrons slowly release energy to produce luminescence. While these materials have demonstrated utility in tumor imaging and cell tracking, their large particle sizes, heavy metal content, and nonbiodegradability pose inignorable biocompatibility risks [30]. Organic afterglow materials, conversely, offer structural tunability, biodegradability, and enhanced biocompatibility [35–37], with applications spanning cancer detection [3,38–40], neuroimaging [41,42], and inflammation monitoring [16,43–45]. Yet, most organic systems depend on visible-light excitation (e.g., white light or 660-nm lasers) [46–49], which restricts tissue penetration depth and limits in vivo signal brightness due to photon scattering and absorption [38,50]. This compromise between excitation depth and afterglow intensity underscores the urgent need for materials that synergize near-infrared excitation—a window with maximal tissue transparency—with high-performance afterglow emission.

Here, we address this challenge by engineering near-infrared-excitable organic afterglow nanoplatform that exploits singlet oxygen (^1^O_2_)-mediated energy transfer to achieve deep-tissue activation and prolonged luminescence. The platform integrates two functional components: (a) a near-infrared absorbing molecule (NAM) that generates abundant ^1^O_2_ under 808-nm excitation as a photosensitizer, and (b) a triplenet-anthracene derivative (TD) that reacts with ^1^O_2_ to produce intense and persistent afterglow. By co-encapsulating NAM and TD into nanoparticles via one-step nanocoprecipitation, we ensure proximity-driven ^1^O_2_-mediated energy transfer while minimizing photobleaching. Through systematic screening of nanoplatform and subsequent optimization, the optimal near-infrared-excitable organic afterglow nanoparticles (NOANPs) were successfully engineered. NOANPs exhibited exceptional afterglow brightness (>10^9^ photons/s at 10 μg/ml) and deep-tissue penetration (3.0 cm ex vivo). Furthermore, the nanoparticles offer a unique capability for tri-modal imaging (fluorescence, white light-activated afterglow, and near-infrared-triggered afterglow), as validated in orthotopic models of pancreatic cancer and glioma. This work overcomes barriers in afterglow imaging by marrying near-infrared excitation with organic material design, paving the way for deep-tissue diagnostics and therapeutic monitoring.

## Results

### Design and characterization of near-infrared-excitable afterglow nanoparticles

**Fig. 1.**
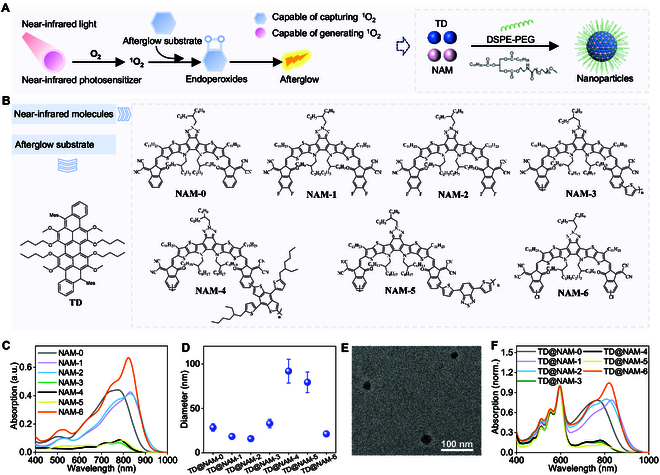
Design and characterization of afterglow luminescent nanoplatform induced by near-infrared light. (A) Schematic illustration of the design of near-infrared-excitable organic afterglow nanoplatform via singlet oxygen (^1^O_2_)-mediated energy transfer. (B) Chemical structures of afterglow substrate and near-infrared molecules. (C) Absorption spectra of different NAM nanoparticles (NAM-NPs). (D) Dynamic light scattering (DLS) size of various TD@NAM nanoparticles (TD@NAM-NPs). Data are presented as the mean ± SD (*n* = 3). (E) Representative transmission electron microscopy image of TD@NAM-NPs. (F) Normalized absorption spectra of various TD@NAM nanoparticles.

To overcome the limitations of the current afterglow systems driven by visible-light pre-irradiation, we engineered a nanoplatform that leveraged singlet oxygen (^1^O_2_)-mediated energy transfer for near-infrared-driven afterglow luminescence. The system integrated two functional components: (a) an electron-rich TD, synthesized as the afterglow substrate, and (b) a NAM capable of generating ^1^O_2_ as photosensitizer. Co-encapsulation of TD and NAM into nanoparticles via one-step nanocoprecipitation with 1,2-distearoyl-sn-glycero-3-phosphoethanolamine-*N*-[methoxy (polyethylene glycol)-2000] (DSPE-PEG2000) ensured proximity between the components, enabling efficient energy transfer (Fig. 1A). ^1^H nuclear magnetic resonance spectra (^1^H NMR) and matrix-assisted laser desorption/ionization time-of-flight mass spectrometry (MALDI-TOF MS) confirmed successful synthesis of TD (Fig. 1B and Figs. S1 and S2), while ultraviolet–visible and fluorescence spectra revealed a broad absorption peak at 590 nm and emission at 635 nm (Fig. S3), aligning with its role as a visible-light afterglow emitter.

Seven NAMs (NAM-0 to NAM-6) were screened as potential afterglow initiators (Fig. 1B). ^1^H NMR and MALDI-TOF MS confirmed successful synthesis of NAMs (Figs. S4 to S14) [51–55]. All NAMs exhibited strong near-infrared absorption [720 to 780 nm in tetrahydrofuran (THF); Fig. S15]. These derivatives were converted to water-soluble nanoparticles by DSPE-PEG2000. Notably, NAM-based nanoparticles exhibited redshifted absorption (Δλ = 15 to 100 nm; Fig. 1C and Fig. S16), which enhanced photon capture at 808 nm, making them well suited for 808-nm laser irradiation.

Co-encapsulation of these NAM and TD (TD:NAM = 100:100, μg:μg) via DSPE-PEG2000 yielded water-soluble nanoparticles (denoted as TD@NAM-NPs) with hydrodynamic diameters of 15 to 95 nm (Fig. 1D and Fig. S18) and spherical morphology (Fig. 1E). Moreover, those nanoparticles showed typical absorption spectra of NAM (peak at around 800 nm) and TD (peak at 590 nm) (Fig. 1F).

### Screening nanoparticles with optimal afterglow performance and studying afterglow luminescence mechanism

**Fig. 2.**
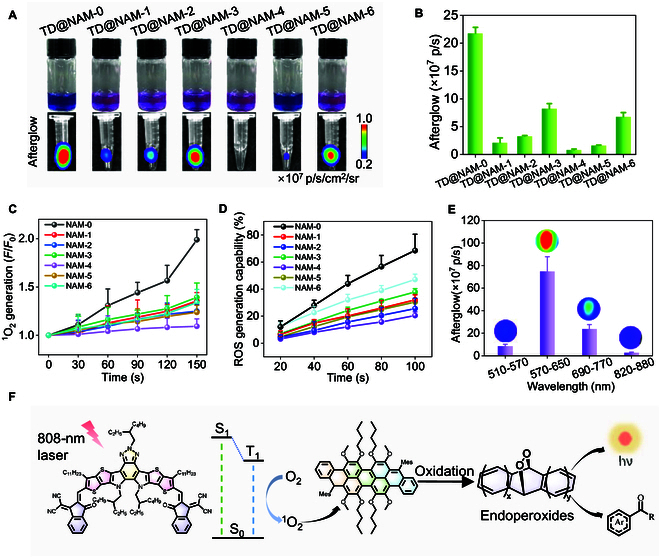
Screening nanoparticles with optimal afterglow performance and studying afterglow luminescence mechanism. (A) Photographs and afterglow images of different TD@NAM-NPs. (B) Quantification of afterglow intensities from (A). (C) Plotting *F*/*F*_0_ versus 808-nm laser irradiation time, using SOSG as indicator for ^1^O_2_ generation. (D) ROS generation ability of various NAM-NPs with different time of 808-nm laser irradiation. (E) Afterglow emission of TD@NAM-0-NPs, obtained through different channels. (F) Schematic diagram of the near-infrared-driven afterglow mechanism. h*ν*, photon energy. Data in (B) to (E) are presented as the mean ± SD (*n* = 3).

The afterglow performance of the nanoparticles is governed by three key factors—^1^O_2_ generation efficiency, ^1^O_2_ capture ability, and fluorescence quantum yield [26]. Among these, the ability to generate ^1^O_2_ plays an important role in determining afterglow intensity. Afterglow intensity, measured via in vivo imaging system (IVIS) imaging post-near-infrared excitation (808 nm, 50 mW/cm^2^, 30 s), revealed TD@NAM-0-NPs as the top performer, emitting 2.17 × 10^8^ photons/s—27.1-fold brighter than the weakest candidate (Fig. 2A and B). This superiority correlated with NAM-0’s exceptional ^1^O₂ generation, validated by singlet oxygen sensor green (SOSG) fluorescence (Fig. 2C and Fig. S19) and 1,3-diphenylisobenzofuran (DPBF) degradation assays (Fig. 2D and Fig. S20). NAM-0-NPs exhibited the fastest SOSG endoperoxide (EPO) formation rate (*F*/*F*_0_ = 1.99 after 180-s irradiation) and 47% DPBF degradation efficiency (after 100-s irradiation), confirming robust reactive oxygen species (ROS) production. In contrast, TA@NAM-5 and TA@NAM-4 exhibit poor ^1^O_2_ generation ability, consistent with their weak afterglow signals.

Spectral alignment between TD’s emission (635 nm) and the DsRed/Cy5.5 detection channels (570 to 770 nm) minimized signal loss (Fig. 2E and Fig. S3), while nanoparticle confinement prevented the outward diffusion of ^1^O_2_. Theoretical calculations for NAM-0 revealed small energy gaps (Δ*E*_S1-T1_ = 0.3354 eV, Δ*E*_S1-T2_ = 0.7038 eV), which are favorable for ^1^O_2_ generation (Fig. S21) [56]. To investigate how the TD captures ^1^O₂ and forms luminescent species, we performed MALDI-TOF MS analysis using a H₂O₂/Na₂MoO₄ system to generate ^1^O₂. Characteristic peaks corresponding to cycloaddition products between TD and ^1^O₂ were detected (Fig. S22), indicating the formation of unstable EPOs. Furthermore, incubation of TD-NPs with ^1^O₂ led to markedly enhanced luminescence compared to phosphate-buffered saline (PBS), H₂O₂, or Na₂MoO₄ alone (Fig. S22) [49]. The afterglow mechanism consists of three phases: (a) Upon 808-nm laser irradiation, the electrons of NAM are excited from the ground state (S_0_) to the singlet state (S_1_), followed by intersystem crossing (ISC) to the triplet state (T_1_). The electrons in the T_1_ release energy and convert the surrounding ^3^O_2_ to ^1^O_2_ in the process of returning to the ground state. (b) ^1^O₂ diffuses into the afterglow substrate TD to form EPOs via cycloaddition. (c) EPOs underwent O–O bond cleavage and decompose, directly emitting photons via chemically initiated electron exchange luminescence (CIEEL) (570 to 770 nm; Fig. 2F).

### Rational optimization of near-infrared-triggered afterglow for TD@NAM-0-NPs

**Fig. 3.**
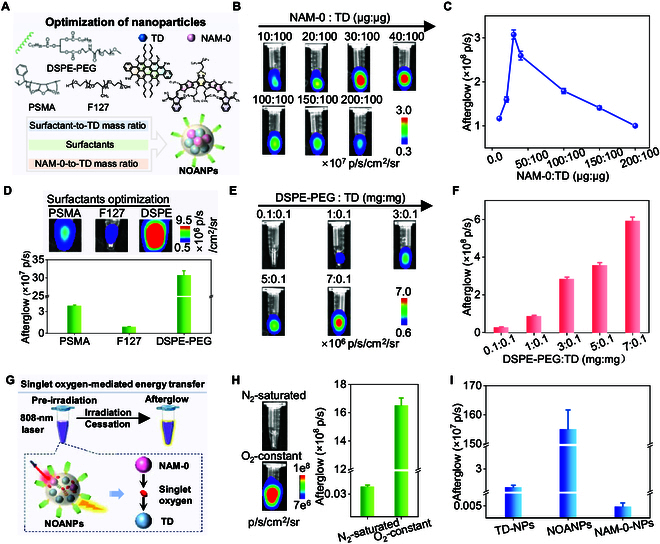
Rational optimization of near-infrared-triggered afterglow for TD@NAM-0-NPs. (A) Illustration of the synthesis optimization of TD@NAM-0-NPs. (B and C) Afterglow images (B) and their corresponding intensities (C) of TD@NAM-0-NPs with different NAM-0:TD ratio. (D) Afterglow images and intensities of TD@NAM-0-NPs encapsulated with various surfactants. (E and F) Afterglow images (E) and their corresponding intensities (F) of TD@NAM-0-NPs with different DSPE-PEG:TD ratio. (G) Schematic representation of singlet oxygen-mediated energy transfer. (H) Afterglow images and intensities of NOANPs under N_2_-saturated or O_2_-constant conditions. (I) Afterglow intensities of TD-NPs, NOANPs, and NAM-0-NPs. Data are presented as the mean ± SD (*n* = 3).

To maximize the afterglow performance of TD@NAM-0-NPs under 808-nm excitation, we systematically optimized three critical parameters: the NAM-0-to-TD mass ratio, surfactants, and the surfactant-to-TD mass ratio (Fig. 3A). The NAM-0:TD ratio directly governs the balance between near-infrared-driven ^1^O₂ generation by NAM-0 and its subsequent trapping by TD to produce afterglow. At suboptimal ratios (NAM-0:TD < 30:100), insufficient ^1^O₂ generation limits luminescence intensity, while excess NAM-0 (NAM-0:TD > 30:100) likely induces aggregation or self-quenching. The optimal NAM-0/TD ratio of 0.3 (NAM-0:TD = 30:100) achieved a peak afterglow intensity of 3 × 10^8^ photons/s 3-fold brighter than the intensities at ratios of 0.1 (NAM-0:TD = 10:100) or 2.0 (NAM-0:TD = 20:100) (Fig. 3B and C). This balance ensures maximal energy transfer efficiency while maintaining colloidal stability.

Surfactant choice profoundly influences afterglow brightness, as it dictates nanoparticle dispersion and molecular packing. DSPE-PEG2000-coated nanoparticles outperformed styrene maleic anhydride copolymer (PSMA)- and Pluronic-F127-stabilized counterparts by 8× and 30×, respectively (Fig. 3D). DSPE-PEG2000’s long PEG chains likely enhance aqueous stability and prevent ^1^O₂ diffusion losses [57], whereas PSMA’s shorter chains and F127’s bulkier structure may hinder NAM-0-TD proximity. Further optimization of the DSPE-PEG/TD ratio to 70 (7 mg DSPE-PEG:0.1 mg TD) maximized afterglow output (3 × 10^8^ photons/s) (Fig. 3E and F). Finally, we determined the optimal ratio parameters (TD: 100 μg, NAM: 30 μg, DSPE-PEG: 7 mg) for synthesizing TD@NAM-0-NPs (NOANPs) and utilized them in subsequent experiments.

The 400-fold afterglow enhancement under normoxic versus nitrogen-saturated conditions indicates ^1^O₂’s indispensable role in the energy transfer cascade (Fig. 3G and H). Control experiments with TD-NPs (TD alone) and NAM-NPs (NAM alone) exhibited negligible afterglow (<1% of NOANPs; Fig. 3I), underscoring the necessity of synergistic NAM-to-TD energy transfer. The 32,714-fold brightness difference between NOANPs and NAM-NPs further highlights the unique ability of TD to capture ^1^O₂ to produce EPOs and release energy as visible luminescence.

### Rational optimization of imaging parameter for NOANPs in solution

**Fig. 4.**
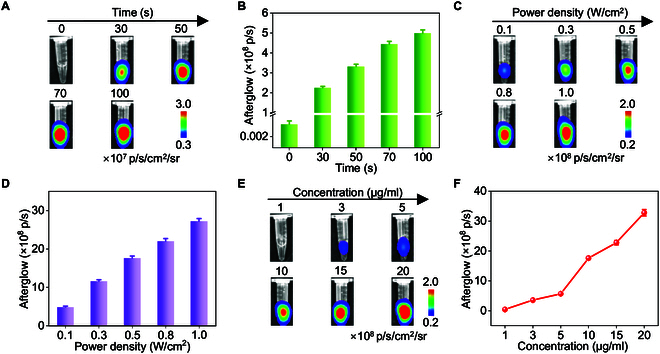
Rational optimization of excitation and imaging parameters for NOANPs in solution. (A and B) Afterglow images (A) and their corresponding intensities (B) of NOANPs under the irradiation of 808-nm laser with different times. (C and D) Afterglow images (C) and intensities (D) of NOANPs under the irradiation of 808-nm laser with different power densities. (E and F) Afterglow images (E) and intensities (F) of NOANPs at different concentrations. Data are presented as the mean ± SD (*n* = 3).

To balance afterglow intensity with biosafety for in vivo applications, we systematically optimized excitation parameters for NOANPs. Afterglow signal of 5 μg/ml NOANPs became stronger with the increase of 808-nm laser irradiation time (0 to 100 s; Fig. 4A and B) and reached 3.314 × 10^8^ photons/s at 50 s (0.7-fold versus 100 s). While prolonged excitation (100 s) marginally enhanced intensity (2.2-fold versus 30 s), the 50-s threshold minimized potential photothermal damage while ensuring robust signal generation [58]. Similarly, afterglow intensity increased with laser power (0.1 to 1.0 W/cm^2^; Fig. 4C and D), yet a pragmatic 0.5 W/cm^2^ setting was selected to avoid tissue overheating while achieving >70% of peak output [59]. Concentration-dependent studies revealed a direct correlation between nanoparticle density (1 to 20 μg/ml) and afterglow intensity (Fig. 4E and F), with 10 μg/ml yielding exceptional afterglow brightness greater than 10^9^ photons/s (1.76 × 10^9^ photons/s).

NOANPs exhibited sustained afterglow emission for >30 min post-excitation, decaying to 3.5 × 10^6^ photons/s after 60 min (Fig. S23). This excellent persistent afterglow emission is due to the spatial limitations of NAM-0 and TD in the DSPE-PEG2000 matrix, thereby minimizing ^1^O₂ diffusion loss.

### Deep-tissue imaging in tissues

**Fig. 5.**
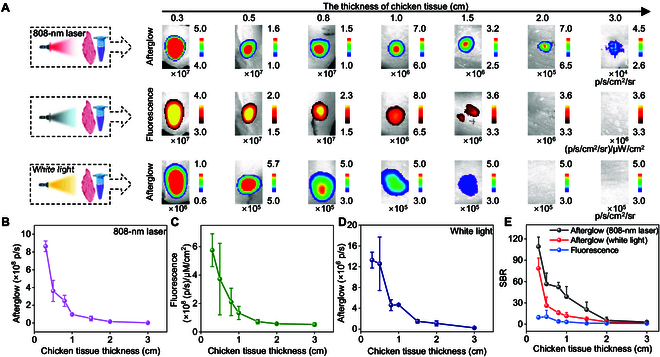
In vitro penetration depth experiments of NOANP afterglow luminescence induced by near-infrared light. (A) The 808-nm laser excitation afterglow images (top), fluorescence images (middle), and white light excitation afterglow images (bottom) of NOANPs covered with chicken tissue of different thicknesses. (B to D) Quantification of 808-nm laser excitation afterglow intensities (B), fluorescence intensities (C), and white light excitation afterglow intensities (D) from (A). (E) SBR for 808-nm laser excitation afterglow, fluorescence, and white light excitation afterglow from (B) to (D). Data are presented as the mean ± SD (*n* = 3).

Chicken breast tissue penetration experiments validated NOANPs’ superiority over white light-activated systems (Fig. 5E and F). Due to the afterglow luminescence property of the nanoparticles with 808-nm excitation, it is beneficial to increase the tissue penetration depth. To verify the improved tissue penetration depth, we compared the penetration depth under white light excitation, 808-nm excitation, and fluorescence excitation. The 808-nm excitation exhibits a distinct afterglow signal at 3 cm. However, white light excitation and fluorescence imaging showed the background signal at more than 1.5 cm. Therefore, the afterglow emitted by 808-nm excitation significantly enhances the tissue penetration depth. Since afterglow imaging has a high imaging SBR, we further compared their SBRs. The afterglow triggered by near-infrared (NIR) maintains an SBR of 2.9 at 3.0 cm, which exceeds the afterglow induced by white light (SBR = 1.1). This advantage stems from the absence of autofluorescence during afterglow acquisition with the 808-nm laser.

### Subcutaneous tumor and deep-tissue orthotopic tumor imaging by near-infrared activated afterglow

The dual-wavelength strategy (near-infrared excitation/visible emission) synergistically enhances penetration depth. Prior to in vivo application, NOANPs demonstrated exceptional stability across physiological buffers [PBS, Dulbecco’s modified Eagle’s medium (DMEM), RPMI 1640], with hydrodynamic diameters remaining nearly unchanged over 24 h (Fig. S24). This stability, attributed to the DSPE-PEG2000 coating, ensures colloidal integrity in biological environments. Furthermore, afterglow and fluorescence signals remained roughly consistent across buffers (Fig. S25), underscoring robustness for diverse biological applications. Confocal imaging using 2′,7′-dichlorofluorescin diacetate (DCFH-DA) and 5,5′,6,6′-tetrachloro-1,1′,3,3′-tetraethylimidacarbocyanine iodide (JC-1) staining showed that, although NOANPs generate ROS under 808-nm laser irradiation, they do not induce significant mitochondrial dysfunction (Figs. S26 and S27). Moreover, cytotoxicity assay revealed >88% viability in 4T1 cells at concentration of NOANPs up to 200 μg/ml (Fig. S28), confirming its excellent biocompatibility and paving the way for its safe in vivo application.

**Fig. 6.**
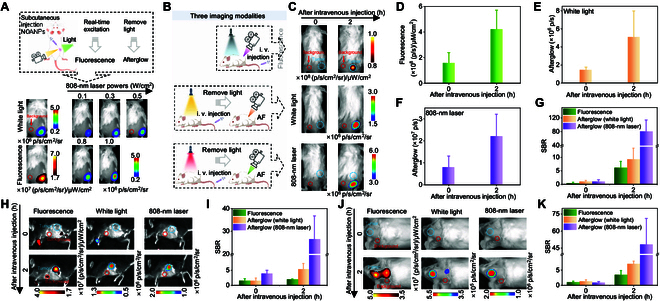
Subcutaneous tumor and deep-tissue orthotopic tumor imaging by near-infrared activated afterglow. (A) Three imaging modalities (afterglow modality under an 808-nm laser pre-irradiation, under white light pre-irradiation, and fluorescence modality) of subcutaneous injection in healthy mice. (B) Schematic representation of the three imaging modalities of subcutaneous 4T1 tumors. (C) Images of three imaging modes of subcutaneous tumor mice at different times after intravenous injection of NOANPs. (D to F) Quantified intensities for fluorescence images (D), afterglow images under an 808-nm pre-irradiation (E), and white light pre-irradiation (F). (G) The SBR of the three imaging modalities was quantified from (D) and (E). (H) Three imaging modality images of mice with orthotopic pancreatic tumors at different times after intravenous injection of NOANPs. (I) SBR quantified from (H). (J) Three imaging modality images of mice with orthotopic brain gliomas at different times after intravenous injection of NOANPs. (K) SBR quantified from (J). Data are presented as the mean ± SD (*n* = 3).

Due to the good biocompatibility, NOANPs were further validated for in vivo imaging. First, we investigated the effect of different powers on the luminescence. Initial subcutaneous injection of NOANPs in healthy mice validated power-dependent afterglow emission under 808-nm excitation (0.1 to 1.0 W/cm^2^), mirroring the solution-phase trend of luminescence enhancement with the increase of power (Fig. 6A and Fig. S29). Next, we performed subcutaneous tumor imaging. Benefiting from the nature of near-infrared light excitation of NOANPs, afterglow triggered by 808-nm laser (0.5 W/cm^2^) exhibited a higher SBR in 4T1 tumor-bearing mice compared to white light afterglow and fluorescence (Fig. 6B to G). This superiority stems from near-infrared light’s deep penetration and absence of autofluorescence, enabling precise tumor delineation. Time-dependent accumulation via the enhanced permeability and retention effect, with SBR reaching 80.8 at 2 h post-injection (Fig. 6G), highlights NOANPs’ capacity for dynamic tumor monitoring. To evaluate the in vivo biodegradability of NOANPs, nanoparticles were intravenously injected into healthy mice. Afterglow signals from major organs were recorded at various time points. Strong afterglow signals were observed in all major organs 2 h after NOANP injection, which gradually decreased to background levels by day 20, indicating effective in vivo biodegradation (Fig. S30).

To address the critical need for deep-tumor detection, the imaging ability of NOANPs was evaluated in the in situ pancreatic cancer and glioblastoma multiforme (GBM) models. The mouse pancreatic cancer model was established by pancreatic in situ implantation of Pan02 cells, and the mouse GBM model was established by implanting C6 cells into the brain. After 1 to 2 weeks, pancreatic and brain tissues from different mouse models were collected and histologically verified through hematoxylin and eosin staining to confirm the presence of tumor lesions (Figs. S31 and S32). In the pancreatic tumor model, obvious afterglow and fluorescence signals were observed 2 h after intravenous injection of NOANPs, and the SBR of 808 nm-induced afterglow reached 26.4 at 2 h, which was higher than that of white afterglow and fluorescence (Fig. 6H and I and Fig. S33). For the challenging deep GBM, obvious fluorescence and afterglow signals were also observed 2 h later, and the SBR of 808 nm-induced afterglow of NOANPs was 48.38 (Fig. 6J and K and Fig. S34), which was higher than that of white light and fluorescence. These results underscore the platform’s ability to overcome photon attenuation in dense tissues, a limitation of conventional visible-light systems.

## Discussion

The NOANPs represent a great advancement in afterglow imaging by synergizing near-infrared excitation with organic afterglow chemistry to overcome the penetration depth of excitation light. Conventional afterglow systems, constrained by visible-light excitation (400 to 700 nm), suffer from limited tissue penetration (<1 cm) and autofluorescence interference [38]. By contrast, the 808-nm excitation wavelength of NOANPs exploits the near-infrared-I biological window, enabling deep-tissue activation while minimizing photon scattering. This strategic decoupling of near-infrared excitation and visible-light emission wavelengths circumvents autofluorescence and achieves a 3.0-cm ex vivo penetration depth—3× greater than conventional white light-activated systems.

The nanoparticles’ exceptional performance stems from its rationally designed energy transfer cascade. The near-infrared-photosensitizer NAM-0 generates ^1^O₂ with high yield, while the electron-rich TD rapidly traps ^1^O₂ to form EPOs that emit sustained afterglow (>30 min). Crucially, the DSPE-PEG2000 matrix confines NAM-0 and TD within 28-nm nanoparticles, ensuring proximity-driven energy transfer and suppressing ^1^O₂ leakage. This nanoscale engineering enables NOANPs to deliver bright afterglow (>10^9^ photons/s at 10 μg/ml).

NOANPs’ tri-modal imaging capability (fluorescence, white light-, and near-infrared-triggered afterglow) addresses heterogeneous needs. While fluorescence provides rapid tumor delineation, near-infrared-activated afterglow achieves unmatched specificity in deep tissues, as demonstrated by SBRs in subcutaneous tumors and gliomas higher than white light or fluorescence modes. Moreover, it is important to note that the tissue penetration depth of afterglow luminescence is closely related to the type of the pre-irradiation light source. Generally, longer excitation wavelengths lead to deeper tissue penetration of the afterglow luminescence [14].

Looking ahead, the platform’s modular design invites expansion into theranostics. Conjugating TD@NAM-NPs with targeting ligands (e.g., cRGD for α_v_β_3_-positive tumors) could enhance specificity, while integrating chemotherapeutic cargos (e.g., doxorubicin) may enable real-time treatment monitoring via afterglow signal modulation. Furthermore, the NAM-TD energy transfer framework could be adapted to other ROS-responsive substrates, permitting emission wavelength tuning for multiplexed imaging.

By merging near-infrared optics with organic afterglow mechanisms, this work transcends the limitations of prior systems and establishes a new paradigm for precision imaging. The TD@NAM-NPs not only advance fundamental understanding of energy transfer in nanoconfined systems but also open avenues for translation in intraoperative guidance and metastatic surveillance.

## Conclusion

This work establishes a high-performance platform for deep-tissue afterglow imaging through the rational integration of near-infrared-excitable chemistry and organic nanoparticle design. Through systematic optimization, NOANPs with ideal photophysical properties were successfully synthesized. The NOANPs overcome the limitations of conventional afterglow materials by synergizing two critical features: (a) near-infrared-driven activation (808 nm) for enhanced tissue penetration and (b) ^1^O₂-mediated energy transfer for bright emission (>10^9^ photons/s at 10 μg/ml). By co-encapsulating a near-infrared photosensitizer (NAM-0) and a triplenet-anthracene afterglow substrate (TD) within a DSPE-PEG2000 matrix, we achieve proximity-driven energy transfer while suppressing ^1^O₂ diffusion losses—a key advancement enabling ultra-bright emission and low photobleaching. The modular design, where NAM-0 and TD serve as interchangeable ^1^O₂ generator and acceptor units, respectively, provides a generalizable blueprint for tailoring afterglow wavelength and activation parameters. This tunability, combined with the demonstrated 3.0-cm ex vivo penetration depth, positions NOANPs as a versatile tool for imaging deep-seated malignancies.

## Methods

The methods can be found in the Supplementary Materials.

## Data Availability

The datasets used and/or analyzed during the current study are available from the author on reasonable request.

## Supplementary Materials

Supplementary 1 Supplementary Materials Table S1 Schemes S1 and S2 Figs. S1 to S34
