# Supplementary material for: Enhanced Near-Infrared-Excitable Organic Afterglow Nanoparticles for Deep-Tissue Multimodal Imaging via Singlet Oxygen-Mediated Energy Transfer
Source: Research (Wash D C). 2025 Aug 14;8:0834. doi: 10.34133/research.0834 (PMC12352855; doi:10.34133/research.0834)
Supplement: Supplementary 1 — Supplementary Materials Table S1 Schemes S1 and S2 Figs. S1 to S34 [file research.0834.f1.docx]

**Supplementary Materials**

**Enhanced** **Near-Infrared-Excitable Organic Afterglow Nanoparticles for Deep-Tissue Multimodal Imaging via Singlet Oxygen-Mediated Energy Transfer**

*Yuzhen Yu^1†^, Zhe Li^1†^,* *Shiyi Liao^1^, Baoli Yin^1^, Qingpeng Zhang^1^, Jiaqi Fu^1^,* *Cheng Zhang^1*^, Ying Zhou^1*^,* *Guosheng Song^1*^*

^[1]^ State Key Laboratory of Chemo and Biosensing, College of Chemistry and Chemical Engineering, Hunan University, Changsha 410082, China.

^*^Address correspondence to: Cheng Zhang; zhang1993@hnu.edu.cn, Yingzhou; zhouying2021@hnu.edu.cn and Guosheng Song; songgs@hnu.edu.cn.

^†^These authors contributed equally.

**Materials.** All reagents were procured from commercial vendors and used without further purification. The following compounds were supplied by Energy Chemical: 2,5,8,11-tetrabromo-1,6,7,12-tetrabutoxyperylene, CuI, MeONa, N-bromosuccinimide,2-formylphenylboronicacid, tetra(triphenylphosphine) palladium (Pd (PPh_3_)_4_), 1,4-dioxane, 2-mesitylmagnesium bromide, boron trifluoride etherate, and K_2_CO_3_. Pluronic F127 and the polymer poly (styrene-co-maleic anhydride) (PSMA) were obtained from Sigma-Aldrich. 1,2-Dimyristoyl-sn-glycero-3-phosphoethanolamine-N- [methoxy (polyethylene glycol)] (DSPE-PEG, Mw = 2000) was acquired from Shanghai ToYongBio Tech, Inc. Dulbecco’s Modified Eagle Medium (DMEM), RPMI-1640 medium, and fetal bovine serum (FBS) were obtained from Gibco Life Technologies. Dulbecco's phosphate-buffered saline (DPBS) was sourced from Thermo Fisher Scientific. Singlet oxygen sensor green (SOSG) was obtained from Dalian Meilun Biotechnology Co., Ltd., and 1,3-diphenylisobenzofuran (DPBF) was acquired from Shanghai McLean Biochemical Technology Co., Ltd.

**Characterization.** ^1^H nuclear magnetic resonance (NMR) spectra were recorded on a VARIAN 400MHz NMR spectrometer, using tetramethylsilane (TMS) as an internal standard. Mass spectra were obtained via matrix-assisted laser desorption/ionization time-of-flight mass spectrometry (UltrafleXtreme). Transmission electron microscope images were accomplished using a JEM-2100F instrument (JEOL). Dynamic light scattering (DLS) size was carried out on the Malvern Zetasizer Nano ZS90 (Malvern). Absorption spectra were acquired via Shimadzu UV-2600 or UV-3600 plus spectrophotometer. Fluorescence spectra were recorded on HITACHI F4600 or Edinburgh fluorescence spectrophotometer. Afterglow luminescence images and fluorescence images were obtained using an IVIS Lumina XR imaging system (Caliper, U.S.A.). H&E-stained slides were scanned with the Pannoramic MIDI digital slice scanning system. The 808 nm stimulator was purchased from Beijing Kaiplin Optoelectronics Technology Co., Ltd. Product model: K808D02FN-8.000W.

**Synthesis and Characterization of TD and NAM-0**.

**Scheme S1.** Synthetic route for TD.

Synthesis of TD: Compound A was prepared by reported method.^[^[^1^](#_ENREF_1)^]^ Compound A (0.5 g, 0.6 mmol) and dry tetrahydrofuran (THF) (50 mL) were added to a 250 mL three-necked flask under nitrogen. 2-mesitylmagnesium bromide (1 M in THF, 70 mL) was added slowly via syringe. The reaction mixture was stirred overnight at room temperature and monitored by TLC. After quenching with water and extraction, the crude product was used directly in the next step without purification. To a solution of crude product in dry methylene chloride (200 mL), boron trifluoride etherate (80 mL, 256 mmol) was added, turning the yellow solution purple. The reaction was stirred overnight at room temperature. After the reaction was quenched with ice water, K_2_CO_3_ was added to neutralize. The residue was purified by column chromatography using methylene chloride/petroleum ether (1/4, v/v) as the eluent, producing TD as a purple solid (0.2 g, yield: 30.2 %). ^1^H NMR (400 MHz, CDCl_3_) δ 9.92 (s, 2H), 7.62 – 7.29 (m, 6H), 7.16 – 7.12 (m, 4H), 4.19 (s, 6H), 4.12 – 4.03 (m, 4H), 3.63 (s, 8H), 3.31 – 3.26 (q, *J* = 7.0 Hz, 2H), 2.51 (s, 6H), 2.03 (s, 6H), 1.80 (s, 6H), 1.45 – 1.40 (m, 8H), 1.31 – 1.28 (m, 8H), 0.84 (t, *J* = 6.8 Hz, 6H), 0.63 (t, *J* = 7.4 Hz, 6H).

**Scheme S2.** Synthetic route for NAM-0.

Synthesis of NAM-0: Compound B was prepared by reported method.^[^[^2^](#_ENREF_2)^]^ Compound B (0.138g, 0.12 mmol) and 1,1-dicyanomethylene-3-indanone (0.276g, 1.21 mmol), pyridine (1 mL) and chloroform (30 mL) were dissolved in a round bottom flask under nitrogen. Then the mixture was stirred and refluxed overnight. After removing the solvent, the crude product was purified on a silica-gel column chromatography to afford 100 mg of compound NAM-0 in 50% yield as a dark blue solid. ^1^H NMR (400 MHz, CDCl_3_) δ 9.14 (s, 2H), 8.69 – 8.67 (m, 2H), 7.95 – 7.93 (m, 2H), 7.75 – 7.73 (m, 4H), 4.75 (d, *J* = 7.8 Hz, 6H), 3.22 (t, *J* = 7.9 Hz, 4H), 2.34 (p, *J* = 6.3 Hz, 1H), 2.05 (p, *J* = 6.7 Hz, 2H), 1.87 (t, *J* = 7.9 Hz, 4H), 1.53 – 1.25 (m, 44H) 1.00 – 0.9 (m, 24H), 0.73 (t, *J* = 7.5 Hz, 6H), 0.62 (t, *J* = 7.2 Hz, 6H).

NAM-1-NAM-6 were prepared by reported method.^[^[^3-6^](#_ENREF_3)^]^

**Screening Nanoparticles with Optimal Afterglow Performance.** To prepare TD@NAM-NPs containing different afterglow initiators (NAM), different NAM (1 mg/mL, 0.1 mL) (NAM-0 to NAM-6), TD (1 mg/mL, 0.1 mL), DSPE-PEG (10 mg/mL, 0.5 mL) and tetrahydrofuran (THF) (0.3 mL) were mixed into a solution (1 mL) and sonicated for 2 min. The solution was then rapidly injected into ultra-pure water (9 mL), followed by ultrasonic treatment for 8 min. The excess THF was removed using rotary evaporation at 45 °C. Finally, different TD@NAM-NPs solutions were purified by ultrafiltration tube (100 KD, 4000 rpm) and stored at ~ 4°C away from light.

To prepare other nanoparticles, the main procedures were similar with that of TD@NAM-NPs. To prepare different NAM-NPs, different NAM (1 mg/mL, 0.1 mL), DSPE-PEG (10 mg/mL, 0.5 mL) and THF (0.40 mL) were mixed into a solution (1 mL).

Near-infrared light-induced afterglow images were collected in bioluminescence (non-excitation) mode using the IVIS Lumina XR imaging system immediately after the 808 nm laser pre-irradiation. Afterglow imaging parameters: bioluminescence mode; filter activated; collection time: 10 seconds unless otherwise specified; field of view: C. Capture images immediately after irradiation and utilize Living Image 4.0 software to analyze the region of interest (ROI).

To measure afterglow intensity of TD@NAM-NPs, TD@NAM-NPs (5 μg/mL, 200 μL) containing different afterglow initiators (NAM) were pre-irradiated with 808 nm laser (0.5 W/cm^2^) for 30 s. Unless otherwise specified, the TD@NAM-NPs concentration is expressed as the mass concentration of TD.

To detect the generation of ¹O₂ in solution, different NAM-NPs (20 μg/mL) were mixed with SOSG (3.52 μM) and irradiated with 808 nm laser (0.1 W/cm^2^) at different time points for 30 s each time. The fluorescence emission at 525 nm for SOSG (excitation wavelength: 480 nm) were measured. Finally, Fx/F_0_ at different time points was calculated. Fx/F_0_ = the fluorescence intensity of SOSG at 525 nm after irradiation with 808 nm laser / the fluorescence intensity of SOSG at 525 nm without 808 nm laser treatment.

To detect the generation of ROS in solution, different NAM-NPs (20 μg/mL) were mixed with ROS indicator 1, 3-diphenylisobenzofuran (DPBF) (30 μg/mL) and irradiated with 808 nm laser (0.1 W/cm^2^) at different time points for 20 s each time. The absorbance at 420 nm for DPBF were measured. ROS generation was assessed by calculating the percentage of DPBF degradation using the following formula 1:

$$ROS generation capability\left( \% \right)=\frac{\left( A_{M0}-A_{MX} \right)-\left( A_{H0}-A_{HX} \right)}{A_{H0}}\times100 (1)$$

Where, *A_M0_* represents the initial 420 nm absorption of DPBF + different NAM-NPs before irradiation, and A_MX_ represents the 420 nm absorption of DPBF + different NAM-NPs after different irradiation times with the 808 nm laser. (*A_M0_* -*A_MX_*) is the absorption difference at 420 nm of DPBF + different NAM-NPs before and after 808 nm laser irradiation. *A_H0_* represents the initial 420 nm absorption of DPBF + H_2_O before 808 nm laser irradiation. *A_HX_* represents the 420 nm absorption of DPBF + H_2_O after different irradiation times with 808 nm laser. (*A_H0_*-*A_HX_*) is the absorption difference of DPBF + H_2_O at 420 nm before and after 808nm laser irradiation.

To detect the capturing capability of ^1^O_2_ in solution, ^1^O_2_ (1 mM, 20 μL), was added into nanoparticles aqueous solution (180 μL), respectively. Then the samples were tested the fluorescence spectra before and after incubating ^1^O_2_ for 5 min. Furthermore, we assessed ^1^O_2_ capturing capability by calculating the decrease percentage of fluorescence intensity, according to Formula 2.

^1^O_2_$capturing capability (\%)=\frac{(F_{P0}-F_{P1})}{F_{P0}}\times100 (2)$

Where, *F_P0_* was the initial fluorescence intensity value of polymer-based nanoparticles group at maximum fluorescence peak before addition ^1^O_2_, *F_P1_* was fluorescence intensity value of polymer-based nanoparticles group at the maximum fluorescence peak after addition ^1^O_2_. (*F_P0_* - *F_P1_*) was fluorescence intensity difference value of polymer-based nanoparticles group at maximum fluorescence peak before and after addition ^1^O_2_.

To detect the fluorescence quantum yield (*QY*) of in solution, rhodamine B was used as a reference to calculate the fluorescence quantum yield (QY) of nanoparticles in aqueous solution (QY = 89% in ethanol), based on the following Formula 3:

$$QYs (\%)=QYr\frac{F_{s}}{A_{s}}\frac{A_{r}}{F_{r}}\frac{n_{s}^{2}}{n_{r}^{2}}\times100 (3)$$

Where, the *QYs* was fluorescence quantum yield of polymers, *QYr* was fluorescence quantum yield of rhodamine B. *As* was the absorbance of polymers and *Ar* was the absorbance of rhodamine B. *Fs* was the relative integrated fluorescence intensity of polymers and *Fr* was the relative integrated fluorescence intensity of rhodamine B. The *n_s_* was the refractive index of the H_2_O (*n_s_* = 2.33) and the *n_r_* was the refractive index of the EtOH (*n_r_* = 1.36).

To measure afterglow emission band, TD@NAM-0-NPs (10 µg /mL, 200 µL) were pre-irradiated with 808 nm laser (0.5 W/cm^2^, 50 s). Immediately after irradiation, the afterglow signals were collected through four different channels (GFP: 510-570 nm; Dsred: 570-650 nm; Cy5.5: 690-770 nm; ICG: 820-880 nm, acquisition time: 10 s), respectively.

**Rational Optimization of Near-Infrared-Triggered Afterglow in TD@NAM-0-NPs.** To prepare TD@NAM-NPs with different NAM-0: TD ratios, different volumes of 1 mg/mL NAM-0 (0.01, 0.02, 0.03, 0.04, 0.10, 0.15, 0.20 mL), TD (1 mg/mL, 0.1 mL), DSPE-PEG (10 mg/mL, 0.5 mL) and THF were mixed into 1 mL solution.

To prepare TD@NAM-0-NPs with different surfactants, NAM-0 (1 mg/mL, 0.03 mL), TD (1 mg/mL, 0.1 mL), surfactant (F127, PSMA, or DSPE-PEG) (10 mg/mL, 0.5 mL), and THF (0.4 mL) were mixed into a 1 mL solution.

To prepare TD@NAM-0-NPs with different DSPE-PEG: TD ratios, NAM-0 (0.03 mL, 1 mg/mL), TD (1 mg/mL, 0.1 mL), 10 mg/mL DSPE-PEG (0.01, 0.1, 0.3, 0.5, 0.7 mL) and different volumes of THF were mixed into 1 mL solution.

To investigate the effect of NAM-0: TD ratios on afterglow luminescence, TD@NAM-0-NPs (5 μg/mL, 200 μL) with different NAM-0: TD ratios were pre-irradiated by 808 nm laser (0.5 W/cm^2^) for 30 s.

To investigate the effect of surfactant modification on afterglow luminescence, TD@NAM-0-NPs (5 μg/mL, 200 μL) with different surfactants was pre-irradiated with 808 nm laser (0.5 W/cm^2^) for 30 s.

To investigate the effect of DSPE-PEG: TD ratios on afterglow luminescence, TD@NAM-0-NPs (5 μg/mL, 200 μL) with different DSPE-PEG: TD ratios was pre-irradiated with 808 nm laser (0.5 W/cm^2^) for 30 s.

To measure the afterglow intensity of NOANPs under N_2_-saturated or O_2_-constant conditions, NOANPs solution (10 µg/mL) was degassed for 2 min and placed under nitrogen atmosphere for 3 min, or not. Then, those samples were pre-irradiated with 808 nm laser (0.5 W/cm^2^) for 50 s.

To compare the afterglow luminescence of NOANPs, TD-NPs and NAM-0-NPs, NOANPs (10 μg/mL, 200 μL), TD-NPs (10 μg/mL, 200 μL) and NAM-0-NPs (3 μg/mL NAM, 200 μL) were pre-irradiated with 808 nm laser (0.5 W/cm^2^) for 50 s.

**Rational Optimization of Excitation and Imaging Parameter.** To study NOANPs afterglow luminescence with different excitation times, NOANPs (5 μg/mL, 200 μL) was pre-irradiated with 808 nm laser (0.5 W/cm^2^) for different times (0 s, 30 s, 50 s, 70 s, 100 s).

To study NOANPs afterglow luminescence at different excitation powers, NOANPs (5 μg/mL, 200 μL) was pre-irradiated for 50 s with 808 nm laser at different powers (0.1, 0.3, 0.5, 0.8, 1 W/cm^2^).

To study afterglow luminescence of NOANPs at different concentrations, 200 μL NOANPs at different concentrations (1, 3, 5, 10, 15, 20 μg/mL) were pre-irradiated with 808 nm laser (0.5 W/cm^2^) for 50 s.

To study afterglow decay time after irradiation, NOANPs (10 μg/mL, 200μL) were pre-irradiated with 808 nm laser (0.5 W/cm^2^) for 50 s.

**Deep-Tissue Imaging in Tissue.** The afterglow and fluorescence images were collected using an IVIS Lumina XR imaging system under bioluminescence (no excitation) and fluorescence modes, respectively. The afterglow luminescence imaging parameters: Bioluminescence modes; Open filter; Acquisition time: 10 s unless otherwise noted; Field of view: C. The power density of light source was determined by the light intensity meter. The fluorescence imaging parameters: Fluorescence modes; Excitation: 605 nm; Emission: Cy5.5 channel unless otherwise noted; Acquisition time: 1 s; Field of view: C.

To investigate the tissue penetration of white light-induced afterglow, 808 nm laser-induced afterglow, and fluorescence imaging, NOANPs (10 μg/mL, 200 μL) were added to centrifuge tubes and then covered with chicken tissue of varying thicknesses (0.3, 0.5, 0.8, 1.0, 2.0, 3.0 cm). Fluorescence images were collected, or after irradiation with white light (6.6 mW/cm², 5 s) and 808 nm laser (0.5 W/cm², 50 s), afterglow images were immediately captured.

**Cell culture and cellular experiment.** Mouse glioma C6 cells, mouse breast cancer 4T1 cells, and mouse pancreatic cancer Pan02 cells were obtained from the American Type Culture Collection (ATCC). 4T1 cells were cultured in RPMI 1640 medium (GIBCO) with 10% fetal bovine serum (FBS) and 1 % of penicillin/streptomycin in a humidified environment at 37 °C with 5 % CO_2_. C6 and Pan02 cells were cultured in DMEM (GIBCO) with 10% fetal bovine serum (FBS) and 1 % of penicillin/streptomycin under the same conditions.

A conventional methyl thiazolyltetrazolium (MTT) test was used to determine dark cytotoxicity. 4T1 cells were seeded in 96-well plates for 24 h, and then NOANPs (final concentrations: 0, 5, 10, 50, 100, 200 µg/mL) were added to the cell culture medium. Cells were incubated with NOANPs or RPMI-1640 for 24 h in dark condition. Finally, the relative cellular viability was tested via the standard MTT assay and measured by microplate reader (Spectramax iD3).

To assess the intracellular ROS production, C6 cells pre-seeded in 4-well plates were divided into three groups, which were incubated with PBS, NOANPs (50 μg/mL) for 8h, and NOANPs (50 μg/mL) for 4 h followed by 808 nm laser irradiation (50 s, 0.5 W/cm^2^) for another 4 h, respectively. After washing with DPBS for three times, those cells were stained with DCFH-DA (an intracellular ROS staining agent) (10 μM) and Hoechst (5 μg/mL) for 30 min, respectively. After washing by DPBS, the fluorescent emission of DCFH-DA (Ex = 488 nm, Em = 530 nm) from those cells was detected using confocal laser scanning microscope (CLSM) to test intracellular ROS.

To assess mitochondrial membrane potential, C6 cells pre-seeded in 4-well plates were divided into three groups, which were incubated with PBS, NOANPs (50 μg/mL) for 8 h, and NOANPs (50 μg/mL) for 4 h followed by 808 nm laser irradiation (50 s, 0.5 W/cm^2^) for another 4 h, respectively. After being washed with DPBS for 3 times, those cells were stained with 5,5’,6,6’-tetrachloro-1,1’,3,3’-tetraethylimidacarbocyanine iodide (JC-1,10 μM) for 30 min. After washing by DPBS, the fluorescent emission of JC-1 (Ex = 514 nm, Em = 529 nm; Ex = 585 nm, Em = 590 nm) was detected using CLSM. The relative fluorescence intensity of JC-1 was measured by ImageJ software. Statistical significance was executed via one-way analysis of variance (ANOVA).

**Measurement of Toxicity of NOANPs and Luminescent Imaging in Different Media.** To evaluate the biocompatibility of NOANPs in cellular level, 4T1 cells were pre-seeded in 96-well plate and then treated with different concentrations of NOANPs (0, 5, 10, 50, 100, 200 µg/mL) for 24 h in dark condition. The relative cell viability was detected by the standard methyl thiazolyl tetrazolium (MTT) method.

To investigate the luminescence intensity of NOANPs in different media, NOANPs (10 μg/mL, 200 μL) were mixed with various buffer solutions (PBS, DMEM, RPMI-1640), followed by fluorescence imaging or afterglow imaging after 50 s of pre-irradiation with an 808 nm laser (0.5 W/cm^2^).

**Subcutaneous Tumor and Deep-Tissue Orthotopic Tumor Imaging by Near-Infrared Activated Afterglow.** All animal experiments were performed in accordance with relevant laws and regulations and were approved by the Institutional Animal Care and Use Committee of Hunan University (SYXK 2022-0007 (Xiang)). BALB/c female mice, KM female mice and C57 female mice were purchased from Hunan Slaike Jingda Experimental Animal Co., LTD., China. To establish a subcutaneous tumor model, 50 μL of 4T1 cells suspension (2×10^6^) were subcutaneously implanted into the right back of female BALB/c mice. For establishment of orthotopic glioma models, 5 μL of C6 cells suspension (5×10^5^) were implanted into the brain of the KM female mice using stereotaxic apparatus with a mouse adaptor. To establish an orthotopic pancreatic tumor model, 25 μL of Pan02 cell suspension (2×10^6^) were orthotopic implanted into the pancreas of C57 female mice.

The afterglow and fluorescence images were performed using an IVIS Lumina XR imaging system under bioluminescence (no excitation) and fluorescence modes, respectively. The afterglow luminescence imaging parameters: bioluminescence modes; Open filter; Acquisition time: 10 s; Field of view: C. The fluorescence imaging parameters: fluorescence modes; Excitation: 605 nm; Emission: Cy5.5 channel; Acquisition time: 1 s; Field of view: C.

For subcutaneous imaging, NOANPs (300 μg/mL, 50 μL) was subcutaneously injected into the right back of mice. Fluorescence images were acquired, and after pre-irradiation with white light (13.4 mW/cm², 5 s) and different powers 808 nm laser (0.1-1.0 W/cm², 50 s), afterglow images were immediately collected.

For imaging of subcutaneous tumor, NOANPs (800 μg/mL, 200 μL) was intravenously injected into 4T1-bearing mice. Fluorescence images, afterglow images (808 nm laser pre-irradiation, 0.5 W/cm^2^, 50 s; white light pre-irradiation, 13.4 mW/cm^2^, 5 s) were collected at 0 h and 2 h post injection of NOANPs.

For imaging of orthotopic glioma tumor and pancreatic tumor, NOANPs (800 μg/mL, 200 μL) was intravenously injected into orthotopic glioma tumor-bearing mice or orthotopic pancreatic tumor-bearing mice, respectively. Fluorescence images, afterglow images (808 nm laser pre-irradiation, 0.5 W/cm^2^, 50 s; white light pre-irradiation, 13.4 mW/cm^2^, 5 s) were collected at 0 h and 2 h post injection of NOANPs.

**Table S1.** Summary of ^1^O_2_ generation efficiency (NAM-0 to NAM-6), ^1^O_2_ capture ability, fluorescence quantum yield, and afterglow intensity of each nanoparticles (TA@NAM-0 to TA@NAM-6).

**Figs. S1 to S34**

**Fig. S1.** ^1^H NMR spectrum of TD in CD_3_Cl at room temperature.

**Fig. S2.** MALDI-TOF-MS spectrum of TD.

**Fig. S3.** (A - B) Normalized absorption spectrum (A) and normalized fluorescence spectrum (B) of TD in THF.

**Fig. S4.** ^1^H NMR spectrum of NAM-0 in CD_3_Cl at room temperature.

**Fig. S5.** MALDI-TOF-MS spectrum of NAM-0.

**Fig. S6.** ^1^H NMR spectrum of NAM-1 in CD_3_Cl at room temperature.

**Fig. S7.** MALDI-TOF-MS spectrum of NAM-1.

**Fig. S8.** ^1^H NMR spectrum of NAM-2 in CD_3_Cl at room temperature.

**Fig. S9.** MALDI-TOF-MS spectrum of NAM-2.

**Fig. S10.** MALDI-TOF-MS spectrum of NAM-3.

**Fig. S11.** MALDI-TOF-MS spectrum of NAM-4.

**Fig. S12.** MALDI-TOF-MS spectrum of NAM-5.

**Fig. S13.** ^1^H NMR spectrum of NAM-6 in CD_3_Cl at room temperature.

**Fig. S14.** MALDI-TOF-MS spectrum of NAM-6.

**Fig. S15.** (A - G) Normalized absorption spectra of different NAM in THF. (A) NAM-0, (B) NAM-1, (C) NAM-2, (D) NAM-3, (E) NAM-4, (F) NAM-5, (G) NAM-6.

**Fig. S16.** (A - G) Normalized absorption spectra of different NAM-NPs in H_2_O. (A) NAM-0-NPs, (B) NAM-1-NPs, (C) NAM-2-NPs, (D) NAM-3-NPs, (E) NAM-4-NPs, (F) NAM-5-NPs, (G) NAM-6-NPs.

**Fig. S17.** (A - G) Normalized fluorescence spectra of different NAM-NPs in H_2_O. (A) NAM-0-NPs, (B) NAM-1-NPs, (C) NAM-2-NPs, (D) NAM-3-NPs, (E) NAM-4-NPs, (F) NAM-5-NPs, (G) NAM-6-NPs.

**Fig. S18.** (A - G) DLS sizes of different TD@NAM-NPs (TD: NAM=100:100, µg: µg) in H_2_O. (A) TD@NAM-0-NPs, (B) TD@NAM-1-NPs, (C) TD@NAM-2-NPs, (D) TD@NAM-3-NPs, (E) TD@NAM-4-NPs, (F) TD@NAM-5-NPs, (G) TD@NAM-6-NPs.

**Fig. S19**. (A - G) Fluorescence spectra of different NAM-NPs under 808 nm laser irradiation with SOSG as fluorescent probe at different irradiation times. (A) NAM-0-NPs, (B) NAM-1-NPs, (C) NAM-2-NPs, (D) NAM-3-NPs, (E) NAM-4-NPs, (F) NAM-5-NPs, (G) NAM-6-NPs.

**Fig. S20.** (A - G) UV absorption spectra of different NAM-NPs under 808nm laser irradiation with DPBF as indicator at different irradiation times. (A) NAM-0-NPs, (B) NAM-1-NPs, (C) NAM-2-NPs, (D) NAM-3-NPs, (E) NAM-4-NPs, (F) NAM-5-NPs, (G) NAM-6-NPs.

**Fig. S21.** The energy levels and ∆E_ST_ values of the NAM-0.

**Fig. S22.** (A) MALDI-TOF-MS spectrum of EPOs complexes after incubation of TD-NPs with ^1^O_2_ (Na_2_MoO_4_ + H_2_O_2_) for 5 min. (B) Afterglow images of TD-NPs incubated with PBS, Na_2_MoO_4_, H_2_O_2_, or ^1^O_2_ (Na_2_MoO_4_ + H_2_O_2_). (C) Corresponding afterglow intensity in panel B.

**Fig. S23.** The decay of afterglow intensities of NOANPs after irradiation.

**Fig. S24.** (A - H) DLS sizes of NOANPs at 0 h and 24 h in different buffer media (H_2_O, PBS, DMEM, RPMI-1640). (A) 0 h, H_2_O, (B) 0 h, PBS (pH=7.4), (C) 0 h, DMEM, (D) 0 h, RPMI-1640, (E) 24 h, H_2_O, (F) 24 h, PBS (pH=7.4), (G) 24 h, DMEM, (H) 24 h, RPMI-1640.

**Fig. S25.** (A) Fluorescence and afterglow images of NOANPs in different buffer media (PBS, DMEM, RPMI-1640). (B, C) Corresponding fluorescent and afterglow intensities from panel A.

**Fig. S26.** Confocal images of C6 cells treated with PBS, NOANPs, or NOANPs + laser irradiation, and stained with DCHF-DA (blue color indicates nucleus; green color indicates ROS).

**Fig. S27.** (A) Confocal images of C6 cells treated with PBS, NOANPs, or NOANPs + laser irradiation, and stained with JC-1. (B) The corresponding ratio of green/ red fluorescent intensity for JC -1 in panel A (P > 0.05, ANOVA). n.s: no statistically significant differences.

**Fig. S28.** The relatively cellular viability of 4T1 cells incubated with different concentrations of NOANPs (without irradiation).

**Fig. S29.** (A-B) Quantification of afterglow intensity at different 808 nm laser excitation powers (A), the SBR of the three imaging modalities (afterglow modality under an 808 nm laser pre-irradiation, under white light pre-irradiation and fluorescence modality) (B), from imaging of subcutaneous injection in healthy mice (Fig. 6A).

**Fig. S30.** (A) Afterglow images of major organs of healthy mice preirradiated with 808 nm laser at different times after intravenous injection of NOANPs. (B) The quantified intensities for afterglow images of major organs in panel A. The time point before NOANPs injection was defined as 0 hour.

**Fig. S31**. H&E staining image of pancreas slice indicating the existing orthotopic pancreatic tumor.

**Fig. S32.** H&E staining image of brain slice indicating the existing orthotopic brain gliomas.

**Fig. S33.** (A-C) Quantification of fluorescence intensities (A)，afterglow intensities under white light pre-irradiation (B), under 808 nm laser pre-irradiation (C), from images of orthotopic pancreatic tumor (Fig. 6H) .

**Fig. S34.** (A-C) Quantification of fluorescence intensities (A)，afterglow intensities under white light pre-irradiation (B), under 808 nm laser pre-irradiation (C), from images of orthotopic brain glioma (Fig. 6J) .

[1] Wang Y, Guo J, Chen M, Liao S, Xu L, Chen Q, Song G, Zhang X-B. Ultrabright and ultrafast afterglow imaging in vivo via nanoparticles made of trianthracene derivatives. *Nat. Biomed. Eng.* 2025; 9(5):656-670.

[2] Luo M, Zhou L, Yuan J, Zhu C, Cai F, Hai J, Zou Y. A new non-fullerene acceptor based on the heptacyclic benzotriazole unit for efficient organic solar cells. *J. Energy Chem.* 2020; 42:169-173.

[3] Liu S, Yuan J, Deng W, Luo M, Xie Y, Liang Q, Zou Y, He Z, Wu H, Cao Y. High-efficiency organic solar cells with low non-radiative recombination loss and low energetic disorder. *Nat. Photonics*. 2020; 14(5):300-305.

[4] Li Z, Zhu C, Yuan J, Zhou L, Liu W, Xia X, Hong J, Chen H, Wei Q, Lu X, et al. Optimizing side chains on different nitrogen aromatic rings achieving 17% efficiency for organic photovoltaics. *J. Energy Chem.* 2022; 65:173-178.

[5] Xu L, Li Z, Ma Y, Lei L, Yue R, Cao H, Huan S, Sun W, Song G. Imaging carotid plaque burden in living mice via hybrid semiconducting polymer nanoparticles-based near-infrared-II fluorescence and magnetic resonance imaging. *Research.* 2023; 6:0186.

[6] Li Z, Zhang N, Chen H, Peng H, Zou Y. Fine-tuning the photovoltaic performance of organic solar cells by collaborative optimization of structural isomerism and halogen atom. *Adv. Energy Sustainability Res.* 2022; 3(1):2100138.
